# Once-Weekly Injection of Low-Dose Teriparatide (28.2 μg) Reduced the Risk of Vertebral Fracture in Patients with Primary Osteoporosis

**DOI:** 10.1007/s00223-013-9777-8

**Published:** 2013-08-21

**Authors:** Takuo Fujita, Masao Fukunaga, Akira Itabashi, Kiichiro Tsutani, Toshitaka Nakamura

**Affiliations:** 1 Katsuragi Hospital, 2-33-1 Habu-cho, Kishiwada, Osaka 596-0825 Japan; 2 Kawasaki Medical School, 577 Matsushima, Kurashiki, Okayama 701-0192 Japan; 3 Saitama Center for Bone Research, 1785-2 Kubojima, Kumagaya, Saitama 360-0831 Japan; 4 Department of Drug Policy and Management, Graduate School of Pharmaceutical Sciences, The University of Tokyo, 7-3-1 Hongo, Bunkyou-ku, Tokyo 113-0033 Japan; 5 National Center for Global Health and Medicine, 1-21-1 Toyama, Shinjuku-ku, Tokyo 162-8655 Japan

**Keywords:** Teriparatide, Fracture risk reduction, Bone mineral density, Primary osteoporosis

## Abstract

We conducted a randomized, double-blind trial to assess the effect of 28.2 μg teriparatide versus placebo (1.4 μg teriparatide) on reduction of the incidence of vertebral fractures. Individuals enrolled in this study included patients with primary osteoporosis with one to five vertebral fractures and capable of self-supported walking. Attention was focused on incident vertebral fractures, change in bone mineral density (BMD) of the lumbar spine, and safety. A total of 316 subjects participated in the study, which lasted up to 131 weeks. Incident vertebral fractures occurred in 3.3 % of subjects in the 28.2 μg teriparatide-treated group and 12.6 % of subjects in the placebo group during the 78-weeks study period. Kaplan–Meier estimates of risk after 78 weeks were 7.5 and 22.2 % in the teriparatide and placebo groups, respectively, with a relative risk reduction of 66.4 % by teriparatide (*P* = 0.008). Lumbar BMD in the 28.2 μg teriparatide group increased significantly by 4.4 ± 4.7 % at 78 weeks, which was significantly higher than the corresponding data in the placebo group (*P* = 0.001). Adverse events were observed in 86.7 % of individuals in the teriparatide group and 86.1 % of those in the placebo group. In conclusion, weekly injection of a low-dose of teriparatide (28.2 μg) reduced the risk of incident vertebral fractures and increased lumbar BMD.

## Introduction

Daily injection of 20 μg teriparatide, an N-terminal fragment of amino acid sequence 1–34 of parathyroid hormone (PTH), increases bone formation and bone mineral density (BMD) [1], thereby improving the microarchitecture of both trabecular and cortical bone [2–5]. Once-weekly injection of 28.2 μg and 56.5 μg teriparatide has been shown to increase BMD in patients with osteoporosis by increasing bone formation [6]. In the 28.3-μg group, only bone-type alkaline phosphatase increased significantly at 4 weeks. Temporal stimulation of bone formation may affect an increase of BMD and contribute to fracture risk reduction. Moreover, injection of 56.5 μg teriparatide once a week reduced the risk of fracture in patients with primary osteoporosis, including older postmenopausal women and men in the Teriparatide Once-Weekly Efficacy Research (TOWER) trial [7].

Prior to the TOWER trial, a once-weekly teriparatide (28.2 μg) trial for reducing fracture risk was stopped early due to the occurrence of osteosarcoma in rats in a concurrent preclinical trial.

In the present study, we report the effects of a low-dose of teriparatide (28.2 μg), corresponding to one-half of that used in the more recently reported once-weekly teriparatide study [7], for preventing vertebral fractures and increasing BMD in men and postmenopausal women with osteoporosis.

## Subjects and Methods

This was a randomized, double-blind trial to assess the effect of 28.2 μg teriparatide versus placebo (1.4 μg teriparatide) on reduction of the incidence of vertebral fractures over a period of 3 years. A total of 329 patients were randomly allocated at 78 sites. Subjects were supplemented with 400 mg calcium throughout the study period. Men and postmenopausal women with primary osteoporosis with one to five vertebral fractures and capable of self-supported walking were eligible for enrollment. Diagnosis of osteoporosis was made according to the criteria of the Japanese Society for Bone and Mineral Research [8].

Prevalent vertebral fractures at enrollment were subjected to lateral spine X-ray examination of the thoracic and lumbar vertebrae, with quantitative assessment according to the criteria of the Japanese Society for Bone and Mineral Research [9, 10]. Patients with secondary osteoporosis [8]; hypersensitivity, such as bronchial asthma or rashes; hypercalcemia; probable pregnancy; primary hypo- or hyperparathyroidism; past history of drug allergy; severe complications, such as cardiac, renal, or liver dysfunction; mental disease; dementia; or severe deformity of the spine were excluded. Patients who had taken bisphosphonates within 48 weeks prior to entry in the study and those treated with calcitonin, active vitamin D derivatives, vitamin K, raloxifene, sex hormone replacement, or anabolic steroids within the previous 8 weeks were also excluded. Serum levels of vitamin D were not measured; therefore, vitamin D deficiency may have been present in this study.

The protocol, which was carried out in accordance with the ethical standards established in the 1964 Declaration of Helsinki and its later amendments, was approved by the internal human studies review board at each center; and informed consent was obtained from each patient.

### Randomization

Patients who satisfied all of the eligibility criteria were randomly assigned in a 1:1 ratio to receive 28.2 μg teriparatide or placebo (1.4 μg teriparatide). Treatment was assigned by computerized dynamic allocation, which was balanced for age, number of prevalent vertebral fractures, and weight via a central enrollment center. The randomization sequence was created by the same individual responsible for investigational product randomization. Registered subjects visited the clinic every week, and physicians injected in a blinded fashion 28.2 μg teriparatide or placebo.

### Procedures

The primary end point was incident vertebral fractures. Secondary end points were incident fractures other than vertebra and change of lumbar spine BMD. All investigators who evaluated the end points were unaware of the study-group assignments of the subjects. Lateral radiographs of the thoracic and lumbar spine were taken at baseline and at 26, 52, 78, and 102 weeks later or upon termination of the trial. One expert investigator independently evaluated the X-ray pictures of the vertebrae from Th4 to L5. Incident vertebral fractures were defined quantitatively in cases where the anterior, posterior, or middle vertebral height had decreased by 20 % or more from baseline in previously nonfractured vertebrae [9, 10]. Nonvertebral fracture was defined by each physician. Dual-energy X-ray absorptiometric (DXA) measurements of the lumbar spine BMD (posteroanterior projection) were performed at baseline and at 26, 52, 78, and 102 weeks. Lumbar DXA was measured at 37 sites with QDR (Hologic, Bedford, MA) and DPX (GE Healthcare, Fairfield, CT) machines in this study. Technicians at each study site provided quality assurance for the longitudinal adjustment by calibrating each machine with standardized phantoms. All DXA measurements were analyzed centrally by a radiologist (M. F.) who was blinded to treatment-group assignment.

All subjects were questioned about adverse events (AEs) at each visit, and all AEs were analyzed regardless of the investigators’ assessments of causality. Data regarding AEs and adverse drug-related reactions (ADRs) were coded by system organ class or preferred terms from Adverse Drug Reaction Terminology (1996) supervised by Japanese Ministry of Health and Welfare. The sponsor (Asahi Kasei Pharma, Tokyo, Japan) produced the teriparatide, monitored each study site, collected the data, and ensured quality control by source data verification during the study period.

### Statistical Analysis

All randomized patients who received any dose of a drug used in the study were included in the safety analysis, and all randomized patients subjected to drug administration who finished the baseline assessment and at least one postrandomization assessment were included in the efficacy analysis. Analysis of vertebral fracture incidence included subjects who underwent radiography at baseline and at least once during the study period. The incidence of new vertebral fractures was analyzed by a log-rank test stratified by the number of prevalent vertebral fractures at baseline. Kaplan–Meier estimates of new vertebral fracture incidence were calculated each time radiography was performed. Reported *P* values were defined by a two-sided alpha of 0.05.

Two-sided Student’s *t* tests were used to determine the intergroup differences in changes in BMD. The incidence of AEs and ADRs were compared by Fisher’s exact test. Results from spinal radiographs, BMD, and other variables were collected centrally and transferred for statistical analyses. The authors had access to all of the data and take responsibility for the veracity of the analyses.

## Results

This study was started in June 1999. A total of 316 subjects participated in the study (158 in each group), and injections of the test drugs were stopped in March 2002. Thirty-three subjects (22.2 %) in the teriparatide group and 22 subjects (13.9 %) in the placebo group discontinued due to AEs or subject request. The duration of observation in each group after completion of injections is summarized in Table 1. The physician recorded the number of injections as a measure of compliance. The longest observation period was 131 weeks in the placebo group (*n* = 1). The mean length of the observation period was 47.4 weeks in the teriparatide group and 50.8 weeks in the placebo group, with no significant difference between the two groups (*P* = 0.399). Eight subjects in the teriparatide group and 15 in the placebo group were excluded from this analysis because of a lack of data for evaluation, discontinuation of previous treatment before obtaining informed consent, or low BMD associated with nonosteoporotic diseases such as multiple myeloma.

**Table 1:** Duration of observation in the teriparatide and placebo groups

Observation period (weeks)	Teriparatide group		Placebo group		P*
	*n*	%	*n*	%	
≤26	52	32.9	52	32.9	0.625
27–52	44	27.8	33	20.9	
53–78	27	17.1	34	21.5	
79–104	24	15.2	26	16.5	
>104	11	7.0	13	8.2	

The maximum observation period was 131 weeks in the placebo group (*n* = 1)

* *P* values were calculated by *χ* ^2^ test

**Fig. 1:**
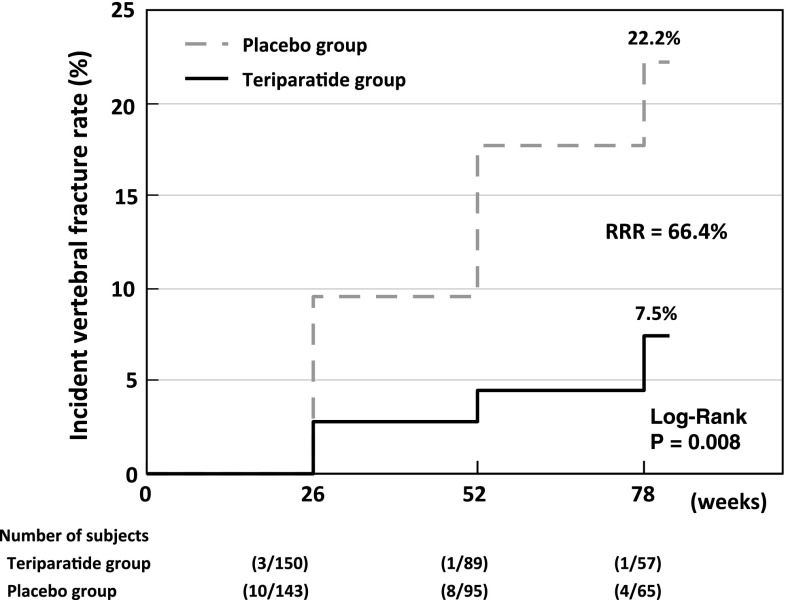
Incidence of new vertebral fractures during the study period (Kaplan–Meier method). *Solid line* teriparatide group, *dashed line* placebo group, *RRR* relative risk reduction

**Fig. 2:**
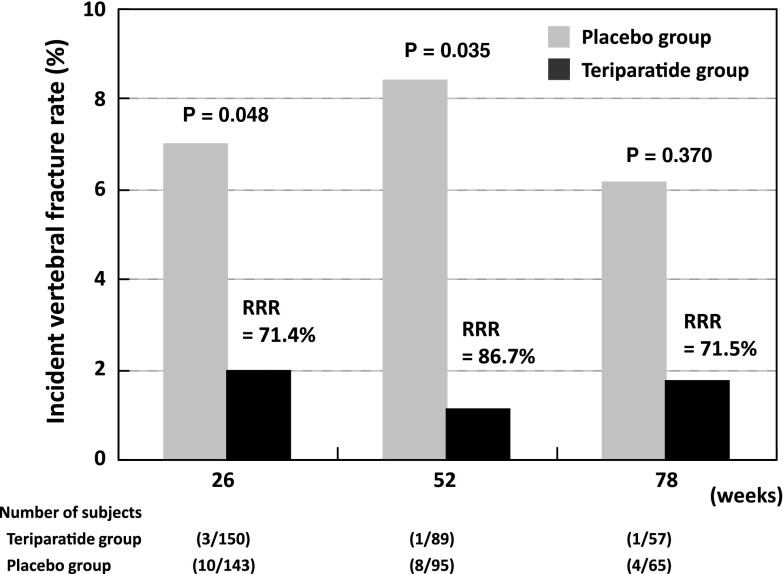
Incidence of new vertebral fractures assessed every 26 weeks. *Black box* teriparatide group, *gray box* placebo group, *RRR* relative risk reduction

There were no statistically significant differences in baseline characteristics between the 28.2 μg teriparatide and the placebo group (Table 2). Incident vertebral fractures occurred in 3.3 % of subjects in the 28.2 μg teriparatide group and 12.6 % in the placebo group during the 78-weeks observation period. Kaplan–Meier estimates of risk after 78 weeks were 7.5 % [95 % confidence interval (CI) 0.3–14.7] and 22.2 % (95 % CI 12.4–32.0) in the teriparatide and placebo groups, respectively, with a relative risk reduction (RRR) of 66.4 % by teriparatide (*P* = 0.008; Fig. 1). Significant reductions in vertebral fracture risk were also observed between the two groups during the first 26 and 52 weeks (Fig. 2).

**Table 2:** Baseline characteristics

Item	Teriparatide group	Placebo group	P*
	(*n* = 150)	(*n* = 143)	
Age (years)	71.6 ± 6.7	71.3 ± 6.9	0.911
Female, cases (%)	144, 92.3 %	134, 93.7 %	0.532
Height (cm)	148.3 ± 6.6	149.4 ± 6.5	0.138
Weight (kg)	50.5 ± 8.1	49.8 ± 7.7	0.452
BMI (kg/m^2^)	23.0 ± 3.2	22.4 ± 3.3	0.093
Lumbar BMD *T* score	−2.80 ± 1.10	−3.02 ± 0.88	0.180
Prevalent vertebral fracture, cases (%)			
1	59, 39.3 %	55, 38.5 %	0.526
2	36, 24.0 %	49, 34.3 %	
3	55, 36.7 %	39, 27.3 %	

Values are mean ± SD or *n*, %

*BMI* body mass index, *BMD* bone mineral density

* *P* values were calculated by *t* test for continuous variables and Chi squared test for binary variables

**Fig. 3:**
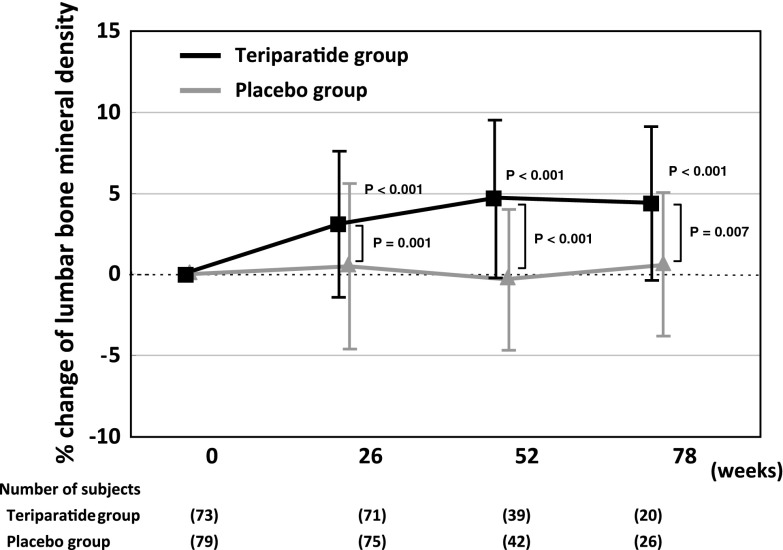
Mean and standard deviation of changes in lumbar bone mineral density over 78 weeks. *P* < 0.05 versus baseline using a paired *t* test and versus the placebo group using an unpaired *t* test

There was no significant difference in the incidence of total nonvertebral fractures at 78 weeks between the teriparatide and placebo groups (3.3 and 5.6 %, respectively; RRR = 49.3 %). Lumbar BMD in the 28.2 μg teriparatide group increased by 3.1 ± 4.5 % at 26 weeks, 4.7 ± 4.9 % at 52 weeks, and 4.4 ± 4.7 % at 78 weeks, which were significantly higher than the corresponding values in the placebo group (*P* < 0.05) (Fig. 3).

Safety data were collected in all of the 316 randomized cases. AEs were observed in 86.7 % of subjects in the teriparatide group and 86.1 % in the placebo group (*P* = 1.000), with no significant differences between the two groups. The incidence of ADRs coded by system organ class in the two groups during the observation period is listed in Table 3. The total rate of ADRs in the teriparatide group was significantly higher than that in the placebo group (24.1 and 9.5 %, respectively; *P* = 0.001). The major ADRs (*n* ≥ 5) in the teriparatide group were nausea (nine cases, 5.7 %) and vomiting (six cases, 3.8 %).

**Table 3:** Adverse drug-related reactions

Classification	Teriparatide group	Placebo group	P*
	(*n* = 158)	(*n* = 158)	
Gastrointestinal system	31	1	<0.001
General	13	2	0.006
Central and peripheral nervous systems	12	2	0.011
Skin and appendages	3	1	0.623
Autonomic nervous system	0	2	0.498
Vision	1	0	1.000
Psychiatric	3	1	0.623
Liver and biliary system	5	3	0.723
Metabolic and nutritional	3	0	0.248
Heart rate and rhythm	1	1	1.000
Respiratory system	0	1	1.000
Red blood cell	0	3	0.246
White cell and reticuloendothelial system	1	1	1.000
Urinary system	3	4	1.000
Resistance mechanism	0	1	1.000

Values indicate the number of incident ADRs reported

*P values were calculated by Fisher’s exact test

## Discussion

In the present study, a significant reduction in the risk of vertebral fracture was observed in the weekly 28.2 μg teriparatide-treatment group compared to the placebo group, with an RRR of 68.4 % over the 78-weeks period. Furthermore, BMD increased significantly to 4.4 % in the teriparatide but not the placebo group. However, the two main effects of weekly 28.2 μg teriparatide in this study were less impressive than those observed with weekly 56.5 μg teriparatide treatment (RRR = 80, 6.7 %; TOWER trial) [7]. The difference in the observed effects on BMD may be explained by the dose of teriparatide. Based on our findings of the differential effects of weekly treatment with 28.2 μg (100 U) and 56.5 μg (200 U) teriparatide on BMD in our previous dose-finding study [6], the 3.6 % increase with 28.2 μg teriparatide in the dose-finding study is comparable to the value obtained in this study. Thus, the difference in BMD increase appears to parallel the antifracture effect.

A simple dose-response relationship alone, however, is insufficient to explain the remarkable effect of a lower dose of teriparatide given weekly in comparison to the effects observed with a higher daily dose [6]. In experimental studies where teriparatide caused simple augmentation of bone formation by osteoblasts via the Wnt pathway [11–14], IGF-I system [15–17], and others, a higher total dose resulted in a proportionally higher response. Therefore, continuous administration providing a higher total dose should theoretically result in a greater response than intermittent administration that inevitably restricts the total dose. However, daily and weekly teriparatide regimens may work by different mechanisms of action. Further prospective studies directly comparing the two dose regimens may clarify this issue.

The actions of teriparatide and intact PTH(1–84) are quite similar but not identical [18]. Upon teriparatide administration, a rather complex relationship exists between the administered teriparatide and secreted endogenous PTH(1–84) whereby teriparatide inhibits the secretion of PTH(1–84) in vivo [19–21] and its release from bovine parathyroid gland in vitro [22]. By recognizing the cumulative actions of PTH and its fragments and taking into consideration the combined effects of the direct action of teriparatide itself and changes to endogenous PTH(1–84), it may be possible to elucidate the yet unknown action of PTH in osteoporosis, thereby facilitating the development of an ideal dosing schedule for teriparatide administration. Shiraki et al. [21] indicated that a single injection of 28.2 or 56.5 μg of teriparatide reduces the level of endogenous PTH(1–84). Suppression of endogenous PTH appears to be greater in the 56.5 μg teriparatide group than in the 28.2 μg group, which is parallel to the effects on BMD and fracture prevention. In addition, excessive levels of endogenous PTH are not necessarily a part of the osteoporosis phenotype but have often been observed in postmenopausal osteoporosis [23] due to vitamin D deficiency, renal insufficiency, or other causes of secondary hyperparathyroidism. Effective suppression of endogenous PTH(1–84) may therefore be one of the factors reducing bone resorption and preventing fracture. Adequate suppression of endogenous PTH(1–84) by teriparatide may be particularly important in severely osteoporotic patients who are frequently in a state of secondary hyperparathyroidism.

The incidence rate of ADRs was 24.1 % in this study, which is similar to that observed with a 28.2 μg dose in our previous dose-finding study (19 %) [6]. This rate is lower, however, when compared to the rate observed with a 56.5 μg (200 U) dose (42 %). The incidence of ADRs may also be affected by the dose of teriparatide.

The first limitation of this study is the unexpected and premature discontinuation of the originally planned trial due to osteosarcoma observed in rats in a concurrent preclinical study. The total number of subjects who were enrolled in the trial, however, was over 140 in each group; and the observation period was not different between the two groups. Moreover, the BMD results in this study confirmed those of our previous dose-finding study [6]. Therefore, it appears reasonable to expect wide and significant implications of the use of low-dose once-weekly teriparatide for increasing BMD. Another limitation is that serum 25-hydroxyvitamin D was not measured; therefore, subjects with secondary osteoporosis due to low levels of vitamin D may have been included in this study. However, measurements of serum Ca, P, and alkaline phosphatase were considered to be adequate for excluding patients with vitamin D deficiency and osteomalacia.

In conclusion, a lower weekly dose of teriparatide (28.2 μg) reduced the risk of incident vertebral fractures and increased lumbar BMD.
